# Neoadjuvant systemic therapy for breast cancer

**DOI:** 10.1093/bjs/znad103

**Published:** 2023-04-28

**Authors:** Alexandra M Zaborowski, Stephanie M Wong

**Affiliations:** Department of Surgery, Royal College of Surgeons in Ireland, Dublin, Ireland; Department of Surgery and Oncology, McGill University Medical School, Montreal, Quebec, Canada; Segal Cancer Centre, Sir Mortimer B. Davis Jewish General Hospital, Montreal, Quebec, Canada

## Introduction

The role of neoadjuvant therapy in the management of breast cancer has evolved dramatically over the past few decades. Preoperative treatment has the potential to downstage tumours and improve breast conservation rates as well as reduce the morbidity of axillary surgery. Furthermore, the *in vivo* sensitivity of the primary tumour to systemic therapy may be assessed at the time of final pathology when treatment is administered before operation. Monitoring of treatment response provides important prognostic and predictive information, with a pathological complete response (pCR) being associated with improved oncological outcomes^1^. Adjuvant therapy may also be affected in patients with residual disease who do not attain a pCR^2–4^. Neoadjuvant therapy for breast cancer may involve chemotherapy or endocrine therapy. The focus of this review is neoadjuvant therapy for the primary tumour in the breast. Axillary management after neoadjuvant therapy has been discussed previously^5–8^ and is beyond the scope of this review.

### Neoadjuvant chemotherapy

Several early landmark trials demonstrated comparable long-term outcomes with neoadjuvant and adjuvant chemotherapy for early and locally advanced breast cancer. The EORTC 10902, NSABP B-18, and NSABP B-27 trials^9–12^ found no significant difference in disease-free survival (DFS) when identical regimens were administered using either approach. Patients who achieved a pCR, however, had superior survival. Pathological response rates to neoadjuvant chemotherapy (NACT) vary according to molecular subtype. A 2020 meta-analysis^13^ of 52 studies including 27 895 patients found the highest rates of pCR in human epidermal growth factor receptor 2 (HER2)-positive tumours at 36.4 (range 17.5–74.2) per cent, and triple-negative breast cancer (TNBC) at 32.6 (20.3–62.2) per cent. Newer trials^14–16^ have reported pCR rates of up to 68 and 65 per cent in HER2+ and TNBC disease, respectively. Hormone receptor (HR)-positive/HER2− tumours consistently demonstrate the lowest pCR rate of 9.3 (5.5–31.3) per cent. Moreover, the association between pCR and long-term outcomes is also less pronounced in this subgroup. In contrast, long-term oncological outcomes are strongly associated with pCR in patients with TNBC and HER2+ tumours, for which the majority of systemic therapy is given in the neoadjuvant setting^1^.

### Surgical decision-making after neoadjuvant chemotherapy

Response to NACT can influence surgical decision-making. Tumour regression may facilitate breast-conserving surgery (BCS) in patients initially thought to require a mastectomy. The randomized CALGB (Cancer and Leukemia Group B) trials of NACT in patients with TNBC and HER2+ tumours found that 42 and 43 per cent respectively of those initially considered to require a mastectomy were suitable for BCS after neoadjuvant therapy^17,18^. Additional prospective trials incorporating platinum-based regimens in TNBC found that 50 per cent of mastectomy-only candidates converted to BCS-eligible after NACT^19^. For patients with HR+ disease, histological subtype can influence breast conservation rates. In a large, single-centre retrospective study, 48 per cent of patients with HR+/HER2− invasive ductal carcinoma who required a mastectomy at diagnosis were eligible for BCS after NACT, compared with only 16 per cent of those with HR+/HER2− invasive lobular carcinoma^20^.

Interestingly, receipt of NACT has also been paradoxically associated with more extensive surgery, despite increasing pCR rates in women who might otherwise have been candidates for BCS^19^. A 2018 meta-analysis^21^ of 16 randomized trials found no association between pCR and BCS rates. There are several possible explanations for this discrepancy, including improved availability of genetic testing, the recommendation to resect all indeterminate microcalcifications at surgery after NACT, multifocal and/or multicentric disease, and the inability to accurately identify pathological response on post-treatment imaging. For example, in HR+/HER2− disease, the non-concentric pattern of tumour regression can make assessment of residual disease difficult^22^. In the CALGB 40603 trial, one-third of patients thought to be ineligible for BCS after NACT had a breast pCR. In the recent UK multicentre NeST study^23^, 36.2 per cent of tumours requiring mastectomy at diagnosis were considered suitable for BCS after NACT. A breast pCR was reported in 34.5 per cent of patients who had a mastectomy and 47.3 per cent who underwent BCS. Surgical practice after NACT varied among centres, with 44 per cent of centres reported excising the original pretreatment tumour footprint, and 55 per cent excising the post-treatment tumour footprint.

Patient preference represents an important aspect of surgical decision-making and may also influence BCS rates after NACT. Results from the BrighTNess trial^19^ showed that only 74 per cent of patients with TNBC who were negative for germline *BRCA1/2* and eligible for BCS opted for this approach. Significant geographical variation was also observed, with women in North America eligible for BCS more likely to undergo unilateral and bilateral mastectomy relative to comparable patients in Europe and Asia. These data highlight the importance of conveying to BCS candidates their accurate risk of residual disease and that locoregional recurrence is altered minimally by more extensive surgery^24^.

### Surgical excision volumes and margins after neoadjuvant chemotherapy

In addition to converting patients who are suitable only for mastectomy at presentation to candidates for BCS, NACT can reduce excision volumes for patients who are already eligible for BCS. The main goal of BCS is to achieve negative margins and a good cosmetic result by removing as little healthy breast tissue as possible. A meta-analysis^25^ of BCS after NACT found significant variation in excision volume. Some studies^26,27^ showed lower excision volumes after NACT , whereas others^28,29^ showed larger or equivalent volumes relative to the upfront surgery population. Pathological response did not always influence excision volume; Volders *et al*.^29^ reported a 5-ml difference in excision volume between patients with no response or a partial response and those with a pCR. The risk of involved margins was, however, higher after NACT compared with primary surgery (24.3 *versus* 10.2 per cent; *P* < 0.001). Successful removal of impalpable breast lesions (clip/fibrosis) after NACT has been demonstrated with both magnetic or radioactive seed localization and wire localization^30^.

Only a few studies have explored the effect of surgical margin width on outcomes after NACT. In a 2018 study by Choi *et al*.^31^, 382 patients treated with NACT underwent breast-conserving therapy, with positive margins noted in 2.1 per cent. Importantly, disease-free and overall survival did not differ for margins of 1 mm or less *versus* more than 1 mm, suggesting that ‘no ink on tumour’ also represents an acceptable margin width for patients treated with NACT. More recent studies including similarly sized cohorts have shown equivalent 5-year locoregional recurrence-free survival rates in patients with narrow (1 mm or less) *versus* wide (over 1 mm) margins (94.2 *versus* 90.6 per cent; *P* = 0.94), supporting this standard-of-care definition for patients treated with NACT, which has already been adopted widely for BCS in the upfront surgical setting^32^.

### Postmastectomy reconstruction after neoadjuvant chemotherapy

Acceptable oncological outcomes have been demonstrated in the setting of immediate breast reconstruction (IBR) after NACT. In a study of 1200 patients with median follow-up of 5.6 years, Wu *et al*.^33^ reported comparable locoregional (3.7 *versus* 3.4 per cent; *P* = 0.83) and distant (17.3 *versus* 18.6 per cent; *P* = 0.68) recurrence rates for skin- or nipple-sparing mastectomy with IBR *versus* total mastectomy without reconstruction. Delayed reconstruction may be preferable, however, to avoid delays to adjuvant treatment in patients with advanced T4 disease who receive NACT. In a retrospective study^34^ of 269 patients, the interval from mastectomy to initiation of adjuvant radiotherapy was significantly longer in those who underwent immediate reconstruction for locally advanced or inflammatory disease. Although this did not appear to affect time to first recurrence, further prospective studies evaluating the relationship between immediate reconstruction and oncological outcomes in this high-risk subgroup are warranted.

In terms of surgical morbidity, immediate reconstruction after NACT is associated with an acceptable complication rate. Several large population-based studies^35–37^ found no association between NACT and implant loss, infection, readmission, or reoperation for reconstructive complications. Similarly, a 2021 meta-analysis^38^ of 17 studies including 3249 patients found that NACT did not increase the risk of complications after IBR (risk ratio (RR) 0.91, 95 per cent c.i. 0.74 to 1.11; *P* = 0.34). A slight increase in implant loss was observed after NACT (RR 1.54, 1.04 to 2.29; *P* = 0.03), which did not delay commencement of adjuvant therapy. Comparison of immediate implant-based reconstruction and autologous flap reconstruction after NACT revealed no significant differences in locoregional recurrence rates or disease-specific survival^39^. Interestingly, some studies have suggested that single-stage direct-to-implant reconstruction after NACT is associated with lower complication rates than two-stage expander/implant-based reconstruction. Although possibly confounded by selection bias, a retrospective study^40^ of 300 patients found a higher rate of implant failure in those who underwent a two-stage procedure after NACT. The interval to adjuvant radiotherapy was also significantly longer in those undergoing a two-stage expander/implant-based reconstructive approach (8.4 *versus* 10.7 weeks; *P* < 0.005).

### Neoadjuvant chemotherapy with immunotherapy

Historically, the systemic treatment of breast cancer relied on multiagent chemotherapy with anthracycline and taxane-based regimens. More recently, several trials have demonstrated the benefit of immune checkpoint blockade in addition to standard chemotherapy in patients with certain subtypes of early-stage breast cancer, particularly TNBC. In the phase II I-SPY2 trial^41^, the estimated pCR rate in patients with early HER2− breast cancer was twice as high in those who received pembrolizumab (anti-programmed cell death protein (PD) 1) in combination with standard NACT than in those who received standard NACT alone. These findings led to the design of the phase III KEYNOTE-522 trial^16^ evaluating the addition of pembrolizumab to NACT in patients with stage II or III TNBC; the proportion of patients who achieved a pCR was higher in the pembrolizumab–NACT group (64.8 *versus* 51.2 per cent). In another trial of the I-SPY2 platform^42^, the addition of durvalumab and olaparib to standard neoadjuvant paclitaxel significantly increased the pCR rate in patients with HER2− breast cancer, irrespective of HR status. Durvalumab and olaparib improved pCR rates from 20 to 37 per cent in HER2− cancers, increasing from 27 to 47 per cent in TNBC, and from 14 to 28 per cent in HR+/HER2− disease. Encouragingly, the pCR rate in patients with Mammaprint® (Amsterdam, Netherlands) ultra-high-risk HR+/HER2− breast cancer was 64 per cent with durvalumab and olaparib compared with 22 per cent with standard paclitaxel.

Encouragingly, the pCR benefit of pembrolizumab appears to translate into improved event-free survival. In the KEYNOTE-522 trial^43^, the estimated event-free survival rate at 36 months was 84.5 per cent in the pembrolizumab–chemotherapy group compared with 76.8 per cent in the placebo–chemotherapy group. These results are similar to those of the GeparNUEVO^44^ trial, in which the addition of durvalumab to NACT for high-risk TNBC improved the 3-year DFS rate from 76.9 to 84.9 per cent (hazard ratio 0.54; *P* = 0.056). These trials have led to a paradigm shift in the management of early high-risk TNBC. Future trials are actively investigating whether a wider population of patients would benefit from neoadjuvant immunotherapy, as well as the synergistic immunomodulatory role of radiotherapy. Using biomarkers or genomic assays to identify subgroups of high-risk patients who do or do not require immunotherapy to achieve a pCR is also an evolving area of research.

### Neoadjuvant endocrine therapy

Substantial tumour downstaging with NACT occurs only in subsets of breast cancer that are chemosensitive owing increased proliferation, gene expression, and molecular subtype. High-grade, ER− and HER2−-amplified tumours respond well to NACT, whereas oestrogen receptor (ER)-positive/HER2− tumours tend to show limited response. Chemotherapy is associated with significant toxicity, limiting its use in frail or elderly patients, and does not necessarily improve distant-disease free survival when used in the adjuvant setting for all postmenopausal women with pN1 HR- positive disease^45^. Neoadjuvant endocrine therapy (NET) represents a well tolerated alternative option for locally advanced ER+ breast cancer, reducing tumour size and improving breast conservation rates. In an early phase II study^45^ comparing NET (anastrozole or exemestane for 12 weeks) and NACT (doxorubicin and paclitaxel for 12 weeks), similar pCR rates (3 *versus* 6 per cent), and a slightly higher rate of BCS (33 *versus* 24 per cent; *P* = 0.058), were observed in the NET group. Chemotherapy was also significantly more toxic, with clinically significant adverse events including neutropenia and neuropathy documented in up to one-third of women. A 2016 meta-analysis^46^ of 20 studies involving 3490 patients found that aromatase inhibitor (AI) monotherapy had BCS rates similar to those of combination chemotherapy (OR 0.65; *P* = 0.07) but with less toxicity.

In terms of NET regimen, data from several studies suggest that AIs are more effective with a better clinical response and BCS rate than tamoxifen in the neoadjuvant setting. The IMPACT RCT^47^ compared 12 weeks of neoadjuvant anastrozole, tamoxifen, or both in postmenopausal patients with ER+ breast cancer. Although no significant differences in clinical or sonographic tumour response were observed among the treatment groups, a higher proportion of patients were deemed suitable for BCS after anastrozole compared with tamoxifen (46 *versus* 22 per cent; *P* = 0.03), suggesting that AIs were superior to tamoxifen. Other trials^48,49^ exploring 12–16 weeks of AI *versus* tamoxifen NET have demonstrated similar results. In the subsequent ACOSOG Z1031A trial^50^, 51 per cent of patients considered eligible for mastectomy only at presentation were suitable for BCS after neoadjuvant AI treatment. Although clinical response rate did not differ significantly across AIs, the clinical response rate was highest following 16 weeks of NET in letrozole-treated (74.8 per cent) and anastrozole-treated (69.1 per cent) patients relative to those receiving exemestane (62.9 per cent). A single-centre study^51^ of 120 patients found a significantly higher pCR rate (17.5 per cent) in patients receiving 12 months of neoadjuvant letrozole, compared with rates of 5 and 2.5 per cent in those who received 8 and 4 months of treatment respectively.

### Predicting response to neoadjuvant endocrine therapy *versus* chemotherapy

The pathological response to neoadjuvant therapy is highly variable. The challenge is to identify patients at diagnosis who are likely to have a meaningful benefit, and to minimize toxicity. A focus of modern adjuvant chemotherapy trials is the evolving role of genomic assays in guiding therapeutic decisions. Several gene expression signatures have been validated to identify patients with early HR+/HER2− breast cancer at low risk of relapse, in whom adjuvant chemotherapy can be omitted safely^52^. The RxPONDER trial^44^ has further expanded the spectrum of patients who can avoid chemotherapy to include postmenopausal women with low-burden nodal disease (1–3 positive axillary lymph nodes) and a 21-gene Oncotype DX^®^ recurrence score of less than 25. Gene expression signatures have also emerged as a potential tool to guide therapeutic decision-making in the neoadjuvant setting. The 21-gene recurrence score is the most well described genomic assay in this context. Several studies have demonstrated higher pCR rates after NACT for HR+/HER2− breast cancer in patients with higher 21-gene recurrence scores. In a meta-analysis^53^ of 1744 patients, the pCR rate was 10.9 per cent in those with high pretreatment recurrence scores (defined as over 25 or over 30) compared with 1.1 per cent in those with a low–intermediate recurrence score (RR 4.47; *P* < 0.001).

Other commercially available gene expression assays include MammaPrint and PAM50. MammaPrint is a 70-gene expression assay that categorizes patients into those at high or low risk of distant recurrence at 5 and 10 years^54^. BluePrint, an 80-gene assay that stratifies tumours into three molecular subtypes (luminal, basal, and HER2), can be used in combination with MammaPrint to characterize luminal tumours as luminal A (low risk) or luminal B (high risk)^55^. Higher pCR rates have been observed in MammaPrint (and BluePrint) high-risk tumours. In the Neoadjuvant Breast Registry Symphony Trial^56^, only 2 per cent of patients with a MammaPrint low-risk tumour had a pCR to NACT, compared with 13 per cent with high-risk tumours (*P* = 0.001).

PAM50 is a 50-gene expression test that classifies breast cancer into five molecular intrinsic subtypes: luminal A, luminal B, HER2-enriched, basal-like, and normal-like. It is also used to estimate a risk of recurrence score that predicts 10-year risk of recurrence, and categorizes tumours into those having a low, intermediate or high risk of distant recurrence^57^. Several studies have shown that, in patients with HR+/HER2− disease treated with NACT, the PAM50 luminal A subtype is associated with the lowest pCR rates. Prat *et al*.^58^ found that both risk of recurrence score (*P* = 0.047) and intrinsic subtype (luminal A *versus* non-luminal A: OR 0.34; *P* = 0.037) were significant predictors of response to NACT. Similarly, in a study by Ohara *et al*.^59^, patients with ER+ disease with the PAM50 luminal A subtype were less likely to achieve a pCR (1.9 per cent) than those with luminal B (9.4 per cent), HER2−enriched (25.0 per cent) and basal-like (12.5 per cent) subtypes (*P* = 0.014). Interestingly, the luminal A subtype defined by immunohistochemistry alone (ER, progesterone receptor, and Ki-67 levels without gene expression) was not associated with lower pCR rates (*P* = 0.51).

More recently, the association between 21-gene recurrence score and response to NET has been investigated. A meta-analysis^60^ of three prospective trials found higher clinical response rates in those with low Oncotype DX recurrence scores. Patients with a recurrence score below 25 (OR 4.60, 95 per cent c.i. 2.53 to 8.37; *P* < 0.001) and those with a recurrence score under 30 (OR 3.40, 1.96 to 5.91; *P* < 0.001) were more likely to achieve a partial response to NET. In the TransNEOS trial^61^ of postmenopausal women with clinically node-negative ER+/HER2− tumours (at least 2 cm in size), the rate of clinical response to neoadjuvant letrozole was 55, 42, and 22 per cent in patients with recurrence scores of under 18, 18–30, and 31 or more respectively. Patients with a low recurrence score were more likely than those with a high score to become suitable for BCS. Conversely, a high recurrence score was associated with disease progression on NET.

Biomarker-driven treatment strategies have also been proposed to guide therapeutic decision-making. An example of such a strategy is the use of proliferative marker Ki-67 to assess response to NET. High Ki-67 expression (over 10 per cent) indicates persistent cell proliferation, thus resistance to treatment, and is associated with poor recurrence-free survival. Data from the POETIC (PeriOperative Endocrine Therapy—Individualising Care) trial suggest that patients with low baseline Ki-67 expression, or those who convert from high to low Ki-67 after 2 weeks of AI therapy, do well with standard adjuvant endocrine therapy alone, whereas those with persistently high Ki-67 expression may require adjuvant chemotherapy^62^. The ALTERNATE phase III clinical trial^63^ randomized postmenopausal women with cT2–4 N0–3 M0 ER+/HER2− cancer to either neoadjuvant anastrozole, fulvestrant, or a combination to identify those with a low risk of disease recurrence. Ki-67 expression after 4 weeks of NET was then used to guide further treatment, such that patients with Ki-67 levels exceeding 10 per cent were switched to NACT. In this group, however, only 4.8 per cent achieved a pCR, suggesting that salvage NACT is unlikely to induce a significant pathological response in these patients, as tumours resistant to NET also appear to be resistant to chemotherapy. More recently, the predictive value of PD-ligand 1 expression has been evaluated. A 2021 meta-analysis^64^ found that high expression was associated with an increased likelihood of achieving a pCR after NACT.

### Omitting surgery in patients with cCR to neoadjuvant systemic therapy

Neoadjuvant systemic therapy has the potential to eradicate the tumour. Pathological response rates have increased dramatically in the past decade, particularly in patients with TNBC or HER2+ disease^14,15^. Achieving a pCR is associated with a low risk of local recurrence and excellent long-term outcomes^1,65^. In patients without residual disease, BCS or mastectomy may have no therapeutic effect. Thus the need for surgery has been questioned, and the concept of adapting management on the basis of response to neoadjuvant therapy has emerged. Efforts to de-escalate treatment in breast cancer have largely focused on axillary surgery. With advances in systemic therapy, omission of breast surgery could be a viable option in appropriately selected patients, minimizing morbidity, avoiding overtreatment, and improving quality of life in survivorship. As ongoing advances in multimodal therapy are likely to further improve pCR rates, non-operative management may represent a potential treatment strategy for an increasing proportion of patients.

A significant factor limiting the implementation of non-operative treatment strategies is the inability to accurately predict the status of residual disease after neoadjuvant treatment. Pathological analysis of the resected specimen remains the standard for diagnosing a pCR or identifying residual disease. Early studies evaluating non-operative management relied on clinical examination and, unsurprisingly, reported high local recurrence rates. Even modern imaging modalities used in combination (mammography, ultrasonography, MRI and PET–CT) are unable to reliably predict a pCR. Imaging with adequate sampling of the residual lesion (or around the remaining clip if a complete radiological response occurs) has been proposed to lower the false-negative rate (FNR). Several small, single-centre pilot studies yielded promising results, but subsequent trials did not reach their primary endpoint^66–68^. Three prospective multicentre trials^69–71^ demonstrated unacceptably high FNRs ranging from 17.8 to 37.0 per cent (*Table 1*). Biopsy technique as well as the number and size of biopsies differed among the studies. Lower FNRs were observed when radiological response and multiple vacuum-assisted biopsies were combined (FNR 5 per cent), or when strict patient selection criteria were applied, including complete/partial clinical response, residual imaging abnormality of 2 cm or less, and at least six vacuum-assisted biopsies taken (FNR 3.2 per cent). Nonetheless, the diagnostic accuracy was insufficient to reliably identify residual disease^69^. In a recent multicentre trial^73^ of 50 patients treated neoadjuvantly, vacuum-assisted biopsies identified a pCR in 31 patients (62 per cent). After omission of surgery, at a median follow-up of 26.4 months, no ipsilateral breast tumour recurrences developed in these 31 patients. Additional larger clinical trials with longer follow-up are needed.

**Table 1:** Studies evaluating false-negative rate of vacuum-assisted biopsy after neoadjuvant chemotherapy

Reference	No. of patients	FNR (%)
Basik *et al*.^72^	98	36.7
Tasoulis *et al*.^71^	166	18.7
van Loevezijn *et al*.^70^	167	37.0
Heil *et al*.^69^	398	17.8

FNR, false-negative rate.

The question of which patients may be suitable for omission of surgery is under intense debate. False-negative vacuum-assisted biopsy results are more likely in older patients with accompanying ductal carcinoma *in situ* (DCIS) in the initial diagnostic biopsy, and multicentric disease on imaging before neoadjuvant therapy. In a prospective multicentre trial^74^ of 398 patients, exclusion of patients with DCIS, multicentric disease, and vacuum-assisted biopsies that did not remove the clip marker, resulted in a FNR of 2.9 per cent. More recently, the concept of intelligent vacuum-assisted biopsy has emerged. Multivariate risk models using machine learning techniques incorporating patient, tumour, and vacuum-assisted biopsy variables have achieved a FNR of 0–1.2 per cent, lower than that for imaging after neoadjuvant therapy, vacuum-assisted biopsy alone, or combinations of both^75,76^. Intelligent vacuum-assisted biopsy alone was also able to accurately identify a pCR in the axilla. Although promising, further prospective confirmatory evidence is needed before implementation in clinical practice.

When considering omission of breast cancer surgery, oncological safety is of utmost importance. Although the concern of leaving residual disease behind is understandable, it is worth remembering that past strategies to de-escalate treatment have not been based on a sensitivity of 100 per cent. The challenge is to determine safe FNRs. It is unknown whether cure rates of a surgery-as-needed strategy are comparable to those of immediate surgery after neoadjuvant therapy. Intensive surveillance may be an acceptable option for selected patients, with surgery reserved for those with recurrence. If long-term survival is similar, some patients may be willing to accept the higher oncological uncertainty in order to avoid a surgical intervention, whereas others may prefer the relative certainty of proceeding with surgery. Patient preference and shared-decision making will be important factors in non-operative treatment strategies.

### Residual disease after neoadjuvant therapy

The association between a pCR and survival is strongest in the most aggressive breast cancer subtypes, that is TNBC and HER2+ tumours, and less pronounced in HR+/HER2− cancer. Achieving a pCR is associated with superior oncological outcomes^13^. Adjuvant chemotherapy after a pCR however, does not appear to improve survival further^13^. Conversely, several RCTs evaluating the use of adjuvant chemotherapy in patients who do not achieve a pCR have demonstrated a survival benefit. The CREATE-X trial^3^ showed improved survival with adjuvant capecitabine in patients with residual TNBC after NACT. In the KATHERINE trial^2^, improved DFS was observed with adjuvant trastuzumab emtansine (T-DM1) *versus* trastuzumab in patients with HER2+ breast cancer who had residual disease after neoadjuvant HER2-directed chemotherapy regimens. The estimated 3-year DFS rate was 88.3 per cent with T-DM1 compared with 77 per cent with trastuzumab alone (hazard ratio 0.50, 95 per cent c.i. 0.39 to 0.64; *P* = 0.001). These trials showed that residual disease alters adjuvant therapy in patients with TNBC and HER2+ tumours. Similarly, the OlympiA^4^ trial reported improved 3-year DFS with adjuvant olaparib in patients with high-risk, HER2− early breast cancer and a germline *BRCA1* or *BRCA2* pathogenic variant, after completion of local treatment and neoadjuvant or adjuvant chemotherapy. In a second interim analysis, 4-year overall survival was also significantly better in the olaparib-treated group, with an absolute survival difference of 3.4 (95 per cent c.i. −0.1 to 6.8) per cent. Ongoing trials evaluating tailored adjuvant therapy in patients with HER2+ cancer and residual disease after HER2-directed neoadjuvant therapy include DESTINY-Breast05 (NCT04622319) and COMPASS-RD (NCT04457596). The former will compare DFS in patients treated with trastuzumab deruxtecan *versus* T-DM1, whereas the latter will compare T-DM1 and tucatinib *versus* T-DM1 alone. Patients with residual disease after neoadjuvant therapy have worse outcomes and urgently require novel therapeutic strategies.

## Data Availability

The data presented in this review are openly available on PubMed, Scopus, and Embase.
